# Characterization and phylogenetic analysis of the complete mitochondrial genome of *Rhinogobius* sp. (Perciformes, Gobiidae)

**DOI:** 10.1080/23802359.2019.1666672

**Published:** 2019-09-20

**Authors:** Tiantian Chen, Mindong Ren, Qingqing Li, Qiming Xie, Shiping Su, Xilei Li

**Affiliations:** ^a^College of Animal Science and Technology, Anhui Agricultural University, Hefei, China

**Keywords:** *Rhinogobius*, Gobiidae, mitochondrial genome, genome characteristics, phylogenetic analysis

## Abstract

The genus *Rhinogobius* was widely distributed in East Asia. In the present study, the complete mitochondrial genome of *Rhinogobius* sp., possible a new species of freshwater goby from Anhui province of China, was sequenced for the first time. Sequence analysis showed that it is 16,511 bp in length with A + T content of 52.3%, consisting of 13 protein-coding genes, 22 transfer RNAs, two ribosomal RNAs, and a control region (CR). Phylogenetic analyses placed *Rhinogobius* sp. in a well-supported monophyletic cluster with other *Rhinogobius* fish and the phylogenetic position of *Rhinogobius* sp. was closer to *Rhinogobius cliffordpopei*.

The genus *Rhinogobius*, known as freshwater goby, is widely distributed in eastern Asia, from Japan to middle and southeastern of China (Thacker 2015; Yu et al. 2016; Zhang and Shen 2019). It is of importance to local peoples as a food fish. Most species of genus *Rhinogobius* are under the threat due to overfishing and habitat destruction, and some species are classed as vulnerable species on the IUCN Red List (http://oldredlist.iucnredlist.org/details/169498/0). In addition, taxonomy within the genus *Rhinogobius* has long been a question debated among scientists (Ogawa and Itoh 2017; Yamasaki et al. 2015). The mitochondrial genome of *Rhinogobius* sp. reported here will promote further understanding of the evolution, taxonomy, and population genetics of this important freshwater goby.

The specimen (*Rhinogobius* sp.) was obtained from Yaodu River, Dongzhi, Anhui, China (117°1′20″E, 30°3′43″N) and was initially identified as *Rhinogobius* sp. based on phenotypic characteristics (Suzuki et al. 2016). Then, the whole muscle was immediately preserved in 95% ethanol and was stored in Laboratory of Aquatic Genetic Resources, Anhui Agricultural University (Voucher No. AAU17072501). Total genomic DNA was extracted using Ezup Column Animal Genomic DNA Kit (Sangon, Shanghai) and stored at −20 °C. The complete mitogenome sequence of *Rhinogobius* sp. was determined using 12 pairs of primers which were designed based on the mitogenome sequence of *Rhinogobius clifordpopei* (Zhong et al. 2018).

The complete mitochondrial genome of *Rhinogobius* sp. is 16,511 bp in length (GenBank accession number: MK288030). It contained 13 protein-coding genes (PCGs), 22 transfer RNA genes (tRNAs), two ribosomal RNA genes (12S and 16S rRNAs), and a control region (CR). The overall base composition was 26.8% of A, 25.5% of T, 16.9% of G, and 30.8% of C. Except for one protein-coding gene *ND 6* and eight tRNAs (*tRNA^Gln^*, *tRNA^Ala^*, *tRNA^Asn^*, *tRNA^Cys^*, *tRNA^Tyr^*, *tRNA^Ser^*, *tRNA^Glu^*, and *tRNA^Pro^*), all other genes are encoded on the heavy strand, which are in accordance with the other teleost mitogenomes (Boore 1999; Xie et al. 2015).

Among the 13 PCGs, all genes used ATG as the start codon except COI, which used GTG. Five PCGs (*ND1*, *COI*, *ATP8*, *ND4L*, and *ND5*) used TAA as stop codon; two PCGs (*ND2* and *ND6*) used TAG as stop codon, whereas five PCGs (*COII*, *COIII*, *ND3*, *ND4*, and *Cytb*) had incomplete stop codon T–– and ATP6 had incomplete stop codon TA–.

To validate the phylogenetic position of *Rhinogobius* sp., we construct the phylogenetic trees of 14 closely related species based on the complete mitogenome sequences using MEGA 7 program (Kumar et al. 2016). *Odontobutis sinensis* was chosen as outgroup. The neighbour-joining tree (Figure 1) showed that *Rhinogobius* sp. first clustered together with *R. cliffordpopei* and formed a monophyly in the genus *Rhinogobius*, and then they constituted a sister-group relationship with other five genera. The present results on the molecular phylogenetic analysis strongly supported that the *Rhinogobius* sp. should be *R. cliffordpopei*. In conclusion, this study also revealed the phylogenetic relationship of the Gobiidae at molecular levels and provided essential nucleotide data for further evolutionary analysis in family Gobiidae.

**Figure 1.**
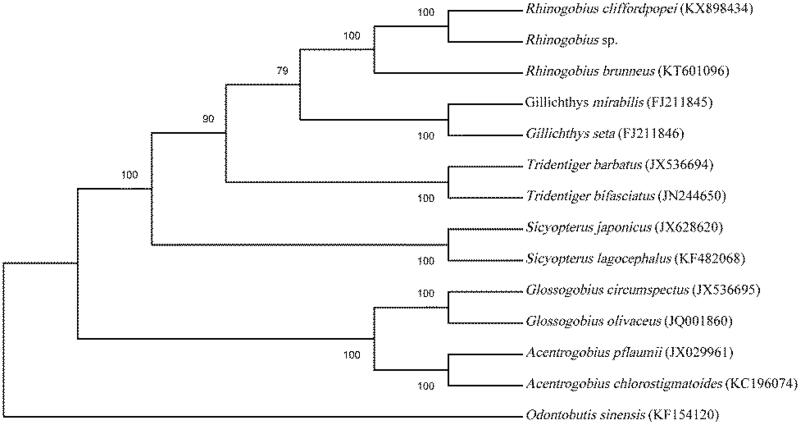
Phylogenetic tree of the Gobiidae based on the NJ analysis of complete mitogenome sequences. Bootstrap values expressed in percentages were shown on the nodes.
